# A Nutraceutical Formula Is Effective in Raising the Circulating Vitamin and Mineral Levels in Healthy Subjects: A Randomized Trial

**DOI:** 10.3389/fnut.2021.703394

**Published:** 2021-09-01

**Authors:** Hatice Zeynep Nenseth, Aparna Sahu, Fahri Saatcioglu, Steven Osguthorpe

**Affiliations:** ^1^Department of Biosciences, University of Oslo, Oslo, Norway; ^2^Turiyan Psyneuronics Pvt. Ltd, Bangalore, India; ^3^Optimal Health Research, Salt Lake City, UT, United States

**Keywords:** nutraceuticals, vitamins, minerals, supplementation, clinical trial

## Abstract

Low levels of nutrient intake are common in industrialized countries. This has negative implications on health and is associated with chronic diseases. Supplementation of vitamins, minerals, and key nutrients to optimal levels may, therefore, be beneficial for individual health and for the health economy. Although the use of supplements has become very common, due to a lack of monitoring, there is very limited data on the efficacy of supplementation with different formulas. In this study, we present the results of a randomized controlled study on the efficacy of a novel formulated nutraceutical, N247, in 250 healthy volunteers aged 26–75 years and a placebo control group (*n* = 35). The broad-spectrum formulation of N247 includes essential vitamins, minerals, and trace elements that are adequately balanced in regard to synergies and related metabolic functions. Moreover, tolerance, safety, and nutrient availability is an important aspect of daily, long-term use of N247. After 3 months of regular N247 use, levels of vitamins and minerals in serum were significantly increased in the N247 group compared with the control group and a placebo group, with excellent compliance rates. Coupled with additional natural ingredients that aim to increase the potency of the nutrients, N247 may represent a novel and beneficial supplement for individuals with nutritional deficiencies.

**Clinical Trial Registration:**https://clinicaltrials.gov/, identifier: NCT04054505.

## Introduction

A large number of laboratory and clinical research suggest that taking dietary supplements may have health benefits and reduce disease burden. Consistently, in those countries where nutritional supplements are commonly used, a greater proportion of the population reaches the estimated average requirements for nutrients (1).

Consumption of dietary supplements has become common in the daily routine of the general public. According to national surveys in the United States, more than half of American adults use at least one form of supplement daily (2). Supplement use increases with increasing age, education and income, and in the same-age groups, and it is more likely among women than men (3, 4). In addition, supplement use has been linked to occupational demands [e.g., (5)]. Supplement users are also more likely to exhibit healthier lifestyle behaviors, such as abstaining from smoking and consuming less alcohol (6, 7). Dietary supplements are also commonly used by health professionals. According to surveys among health professionals, 88% of nurses, 84% of physician assistants or nurse practitioners, 66% of pharmacists, and 72% of trainees reported taking supplements in which multivitamins (60%), calcium (40%), vitamin B (31%), vitamin C (30%), and fish oil (24%) are the top listed (8). Multivitamin/mineral supplements are widely used and easily accessible in pharmacies, health food stores, and online stores. However, there is very limited information on the effectiveness of dietary supplements. A report presented by the National Nutritional Foods Association in the United States showed that the majority of respondents anticipate receiving information about supplement use from their pharmacists (84%) and physicians (80%) (9). Furthermore, health professionals should be informed about supplement usage of their patients to ensure a balanced intake of nutrition as well as to avoid drug-nutrient interactions and potentially toxic levels of some nutrients (2, 10).

Nutritional deficiencies are a widespread incident in industrialized countries due to energy-rich and nutrient-low dietary habits, which has been exasperated during the COVID-19 pandemic (11–15). In addition, the use of certain medications, such as oral contraceptives, can result in micronutrient depletion (16). Thus, in addition to promoting healthier food options and physical activity, dietary supplements are beneficial when an individual has an inadequate or unbalanced diet. Aiming for supplementation at or slightly above the official Recommended Dietary Allowances (RDAs) may be a simple step to mitigate inadequate and unbalanced physiological levels of vitamins, minerals, and trace elements (17). Although RDA is established to meet the average nutrition intake in all healthy persons overall (18), higher doses of individual nutrients in excess of the RDAs may be necessary to achieve adequate levels for a given individual in regard to age, level of physical activity, stress, genetic factors, medication usage, toxic burdens, or disease (19, 20). Therefore, increased intake of vitamins and minerals and key nutrients by supplementation may be beneficial for optimal functioning of multienzyme systems, metabolism, hormonal regulation, and detoxification pathways that are important to maintain well-being and improve health span (21). To achieve this goal, a vitamin, mineral, and a key nutrient supplement was formulated, termed N247, which effectively improved their levels in circulation. In this study, we provide data on its assimilation in subjects of a wide age range.

## Materials and Methods

### N247 Ingredient Profile

The quantities and forms of all nutrients in N247 are adjusted for aiming a high level of tolerance, nutrient bioavailability, and safety with daily, long-term use. Moreover, the broad-spectrum formulation of essential vitamins, minerals, and trace elements in N247 targets synergies and related metabolic functions among these constituents. In contrast to common over-the-counter multivitamins, the higher strength of ingredients in N247 is expected to offer greater nutritional benefits (21–23). The complete list of N247 ingredients and their special forms and amounts per dose are summarized in Table 1. Some of the distinguishing characteristics of N247 are described below.

**Table 1:** Ingredient list, dosage, and special forms of N247.

**Ingredients in N247**	**Amount per packet**	**Source**	**% Daily value**
Vitamin A	7,000 I.U.	50% vitamin A palmitate; 50% natural carotenes	140%
Vitamin C	250 mg	L-ascorbic acid USP	417%
Vitamin D3	1,200 I.U.	cholecalciferol	300%
Vitamin E	250 I.U.	60% d-alpha tocopheryl succinate plus mixed tocopherols d-beta, d-delta, and d-gamma from soy; 40% d-alpha tocopherol acetate	833%
Vitamin K1	37.5 mg	Phytonadione	47%
Vitamin K2	22.5 mg	menaquinone-7**^‡^**	28%
Thiamin	25 mg	thiamin hydrochloride USP	1667%
Riboflavin	15 mg	riboflavin USP	882%
Niacin	60 mg	67% niacinamide USP; 33% niacin USP	300%
Vitamin B6	15 mg	pyridoxine hydrochloride USP	750%
Folic acid	400 mcg****	50% folinic acid (5-formyltetrahydrofolate); 50% L-5-methyltetrahydrofolate	100%
Vitamin B12	50 mcg	50% methylcobalamin; 50% 5-adenosylcobalamin	833%
Biotin	300 mcg	biotin USP	100%
Pantothenic acid	50 mg	d-calcium pantothenate USP	500%
Calcium	100 mg	calcium citrate-malate	10%
Iodine	150 mcg	from kelp	100%
Magnesium	100 mg	magnesium glycinate chelate**^§^**	25%
Zinc	15 mg	zinc glycinate chelate**^§^**	100%
Selenium	200 mcg	selenium amino acid complex	286%
Manganese	5 mg	manganese glycinate chelate**^§^**	250%
Chromium	150 mcg	50% chromium polynicotinate; 50% chromium nicotinate glycinate chelate**||**	125%
Molybdenum	100 mcg	molybdenum glycinate chelate**^§^**	133%
Boron	2 mg	boron aspartate-citrate	^*^
Vanadium	50 mcg	bisglycinato oxovanadium	^*^
Choline	75 mg	choline bitartrate	^*^
Inositol	25 mg	Inositol	^*^
Citrus bioflavonoids	100 mg	85% citrus bioflavonoid complex with a min. of 50% bioflavonoids; 15% hesperidin methylchalcone	^*^
L-Taurine	300 mg	L-Taurine	^*^
Hawthorn (Crataegus pinnatifida Bge.) berry	250 mg	dried berry extract with a min. of 4% vitexin	^*^
ActiVin ^**^Grape seed (Vitis vinifer)	200 mg	dried seed extract with a min. of 80% phenolic content	^*^
CoEnzyme Q10	200 mg	Ubiquinone	^*^
Alpha-Lipoic acid	200 mg	alpha-lipoic acid	^*^
Marine lipid concentrate	1,000 mg		^*^
**Supplying the following Omega-3 fatty acids**			
Eicosapentaenoic acid (EPA)	360 mg^¶^ (330 mg^#^)		^*^
Docosahexaenoic acid (DHA)	270 mg^¶^ (250 mg^#^)		^*^
Acetyl-L-Carnitine hydrochloride	500 mg	Acetyl-L-Carnitine hydrochloride	^*^
L-Glutamine	250 mg	L-Glutamine	^*^
Pyroglutamic acid	100 mg	Pyroglutamic acid	^*^
L-Tyrosine	250 mg	L-Tyrosine	^*^
DMAE	100 mg	dimethylaminoethanol	^*^
Ashwagandha (Withania somnifera)	100 mg	dried extract with a min. of 1.5% withanolides and min. of 1% alkaloids	^*^
Blueberry (Vaccinum corymbosun) fruit	100 mg	dried fruit extract with a min. of 16% chlorogenic acid	^*^
Ginkgo Biloba	80 mg	dried extract with a min. of 24% ginkgo flavone glycosides, min. of 6% terpene lactones and <1 ppm ginkgolic acid)	^*^
Siberian Ginseng (Eleutheroccus senticosus) root	100 mg	dried root extract with a min. of 0.8% ginsenosides	^*^
Vinpocetine	10 mg	Vinpocetine	^*^
Rhodiola Root (Rhodiola rosea)	100 mg	dried root extract with a min. of 2% rosavins, min. of 1% salidrosides	^*^
SerinAid****	330 mg		^*^
Phosphatidylserine	99 mg		^*^
Phosphatidylethanolamine	26 mg		^*^
Phosphatidylcholine	33 mg		
Phosphatidylinositol	13 mg		^*^
Lutein	20 mg		^*^
Zeaxanthin	1,000 mcg		
Others	–	Carboxymethylcellulose sodium, hydroxypropyl methylcellulose, gelatin, cellulose, silicon dioxide, vegetable stearine, glycerin, natural mixed tocopherol vitamin E, gum ghatti, magnesium stearate, natural lemon oil, rosemary extract, natural citrus flavor, stearic acid, cellulose coating, and water	–

*The percentage daily values are according to the Nutrient Reference Values for the USA. Recommendations are for an adult of 18 years or older and based on a 2,000-calorie diet*.

* *Percentage daily value not established. ****Contains MenaQ7™ (NattoPharma, Norway; patents pending)*.

‡ *Amount of active isomers from mixed isomer forms*.

§ *Albion Laboratories, USA. ^**||**^ChromeMate (InterHealth, California, USA; covered by one or more U.S. Patents 4,923,855, 4,954,492, and 5,194,615)*.

¶ *Amounts reported as the weight of the fatty acid compound*.

# *Amounts reported as free fatty acid equivalents by weight in accordance with voluntary CRN Monograph*.

** *ActiVin is a trademark of San Joaquin Valley Concentrates, Inc. ****SerinAid is a registered trademark of Chemi Nutraceuticals*.

*I.U, international unit; USP, United States Pharmacopeia grade*.

#### Vitamin A/Carotenoids

Vitamin A is a fat-soluble vitamin and an antioxidant. It supports skin, hair, and nail growth, visual function, and immune system reproductive health. Both precursors of vitamin A, preformed vitamin A (from animal sources) and provitamin A (from plant-based sources, commonly β-carotene), are present in the N247 formula to achieve a balanced intake of vitamin A. Diabetes and hypothyroidism are often accompanied by insufficient conversion of β-carotene to vitamin A. Therefore, β-carotene supplementation alone may not be enough to support immune cell activation (24). N247 contains retinol, the natural unsaturated cis-form from fish liver oil, that may improve absorption, for example, in the case of compromised gastrointestinal tract function (25). N247 includes mixed, natural-source carotenes of vitamin A palmitate [50% corresponding to 3,500 international unit (I.U.)], and natural carotenes, that is, alpha-carotene, beta-carotene, cryptoxanthin, zeaxanthin, and lutein (50% corresponding to 3,500 I.U.) from *D. salina*.

#### Vitamin C/Bioflavonoids

Vitamin C is a water-soluble vitamin and an antioxidant. It is essential for maintaining healthy skin, blood vessels, bones and cartilage, and for destroying free radicals. It also helps to increase LDL resistance to oxidative stress and contributes to decrease it in erythrocytes (26, 27). In N247, vitamin C is found as l-ascorbic acid United States Pharmacopeia grade (USP), which is a buffered, mineral-bound form of vitamin C, to prevent the gastric irritation caused by the normal acidity of ascorbic acid. In addition, vitamin C activity is accompanied by a concentrated source of citrus bioflavonoids.

#### Vitamin E Complex

Vitamin E complex is a fat-soluble vitamin and an antioxidant. It is important to fight oxidative stress, protect the cell membranes, and regulate platelet aggregation (28). It also prevents glucose-induced lipid peroxidation and protects against stress-induced mucosal lesions (29, 30). N247 contains a natural vitamin E complex in the form of d-alpha-tocopherol succinate and tocopherols of d-beta, d-delta, and d-gamma from soy (60% corresponding to 150 I.U.) and d-alpha-tocopherol acetate (40% corresponding to 100 I.U.).

#### Vitamin D

It is a fat-soluble vitamin that is important for the maintenance of normal levels of calcium and phosphate in the blood by absorption from food and urine, contributing to bone homeostasis and retention of calcium in the bone (31). It is also synthesized in immune cells where it can regulate the adaptive and innate immune responses (32). In N247, vitamin D is present as cholecalciferol USP from natural sources at levels of the recommended daily intake (400 I.U.).

#### Vitamin B Complex

N247 contains all B-complex vitamins. Among these are folic acid (inactive forms as folinic acid and 5-methyltetrahydrofolate), vitamin B6, and vitamin B12 (inactive forms as methylcobalamin and adenosylcobalamin) that maintain adequate levels of methylation and keep the homocysteine levels low (33).

##### Thiamine

Thiamine is a water-soluble vitamin (B1) that is essential for the pyruvate metabolism in the breakdown of nutrients for energy, and it contributes to a healthy nervous system (34). In N247, it is found as thiamine hydrochloride USP.

##### Riboflavin

Riboflavin is a water-soluble vitamin (B2). It participates in a range of metabolic redox reactions that are important for aerobic cell functions, and it acts as an antioxidant reducing oxidative stress (35, 36). It is required to process amino acids and fats and helps to convert carbohydrates to energy or ATP.

##### Niacinamide

Niacinamide is a water-soluble vitamin (B3) and a precursor to the cofactors nicotinamide adenine dinucleotide and nicotinamide adenine dinucleotide phosphate (NADPH), which are essential in the breakdown of carbohydrates, cytoprotective in the central nervous system (37, 38). In N247, it is found as niacinamide USP (67%) and as niacin USP (33%).

##### Vitamin B6

Vitamin B6 is a water-soluble vitamin (pyridoxine). It is a cofactor for many enzymes involved in protein metabolism. It also participates in hemoglobin biosynthesis, glucose, and lipid metabolic processes and is important in immune and nervous system function (39, 40). In addition, it has antioxidant properties (41). In N247, it is found as pyridoxine hydrochloride USP.

##### Folate

Folate is a water-soluble vitamin that acts as a coenzyme in single-carbon transfers in the synthesis of DNA and RNA. It is involved in the conversion of homocysteine to methionine in the synthesis of methyl donors, thereby reducing heart disease risk and production of red blood cells where its deficiency may lead to anemia (42).

##### Vitamin B12

It is a water-soluble vitamin (cobalamin) that is essential for the development and proper functioning of the central nervous system (43). It participates in the remethylation of homocysteine to methionine together with folate and vitamin B6, thereby reducing heart disease risk (44). In N247, it is found as methylcobalamin (50%) and as adenosylcobalamin (50%).

##### Pantothenic Acid

A water-soluble vitamin (also known as B5), pantothenic acid is a precursor of acetyl CoA and is essential for the breakdown of fats, carbohydrates, and proteins to release energy (45). In N247, it is found as d-calcium pantothenate USP.

##### Biotin

Biotin is a water-soluble vitamin (part of the B complex) that acts as a coenzyme and is essential for growth, development, and normal mitochondrial and cellular functions (46).

#### Minerals/Trace Elements

N247 contains mineral and trace elements, such as zinc, magnesium, manganese, and copper as well as chromium and vanadium (in organically bound forms). Iron is not included in N247, as the majority of adults do not need supplemental amounts of iron that may have prooxidant and cytotoxic effects (47).

##### Calcium

Calcium is a mineral that is an important regulator in many biological processes. It is involved in nerve function and muscle contraction, for forming strong bones and teeth, blood clotting, regulating heartbeat as well as fluid balance within cells (48). In N247, it is found as calcium citrate-malate. Please note that since other ingredients of N247 have calcium included as salts, the amount listed for calcium alone may first appear to be low, but in the context of the whole supplement, it is at the RDA level.

##### Iodine

Iodine is a mineral that is essential for triiodothyronine and thyroxine synthesis. Its deficiency leads to hypothyroidism and impaired development of the central nervous system (49). In N247, it is sourced from kelp.

##### Magnesium

This mineral is essential for nucleic acid and protein synthesis, fat and glucose metabolism, oxidative phosphorylation, and neuromuscular excitability (50). In N247, it is found as magnesium glycinate chelate.

##### Zinc

The mineral zinc is an essential part of many enzymes and hormones acting as structural, catalytic, intracellular, and intercellular signaling components (51). It is involved in the integrity and normal development of the immune system, bone formation, and tissue growth (52). In N247, it is found as zinc glycinate chelate.

##### Selenium

This mineral is required for the production of thyroid hormone-metabolizing enzymes and is implicated in immunity and antioxidant defense (53, 54). In N247, it is found as a selenium amino acid complex.

##### Manganese

This mineral is an important component of several enzymes, such as pyruvate carboxylase, lipase, superoxide dismutase, and oxygen-handling enzymes. It is involved in bone formation, regeneration of blood cells, and proper function of the central nervous system (55). In N247, it is found as manganese glycinate chelate.

##### Chromium

The mineral chromium can lower total cholesterol and low-density lipoprotein, enhance insulin sensitivity and glucose metabolism (56). In N247, it is found as chromium polynicotinate (50%) and as chromium nicotinate glycinate chelate (50%).

##### Molybdenum

This mineral is an essential factor of several enzymes, such as nitrogenase, nitrate reductases, sulfite oxidase, and xanthine oxidoreductases among others. Dietary molybdenum has been used to treat iron deficiency anemia and arthritis (57). In N247, it is found as molybdenum glycinate chelate.

##### Potassium

Potassium is a mineral that is essential for enzymatic reactions that take part in protein synthesis, glycogen synthesis, cell growth, and cell division. It regulates fluid balance, blood pressure, and acts as a local mediator of vascular tone in muscle beds (58). In N247, it is found as potassium gluconate.

#### Other Ingredients

In addition to the above vitamins and minerals, several other ingredients are included as described below. These aim to increase the effectiveness of the vitamins and minerals, as well as bringing additional benefits to increase wellness [e.g., (59, 60)].

##### Inositol

This mineral is an important sugar compound of several biological molecules that are involved in cell growth and survival, osteogenesis, and the development and functioning of peripheral nerves (61–63). In N247, it is included for a balanced formulation of neuronutrients which can address mild cognitive impairment (64).

##### Acetyl-l-Carnitine

Acetyl-l-carnitine is important for mitochondrial energy production (65). It also provides neuroprotection in hypoxic stress and improves cognitive impairment (66, 67).

##### l-Glutamine

This is an amino acid that is an essential part of numerous molecules involved, for example, in nucleotide synthesis, NADPH is an antioxidant (68). In addition, astrocyte-derived l-glutamine is a precursor of the neurotransmitter glutamate, carrying an excitatory function, and GABA, having an inhibitory function (69).

##### l-Pyroglutamic Acid

A derivative of glutamate, it is an important component of several proteins and neuropeptides. It can enhance GABA release, which activates the GABA system and cortical acetyl-choline (70).

##### l-Tyrosine

l-Tyrosine is an amino acid that is an important precursor of catecholamines. Changes in bioavailability in the brain can affect the production of neurotransmitters, such as dopamine and norepinephrine. It can also acutely reverse the effects of cognitive decline in response to physical stress (71, 72).

##### Dimethylaminoethanol

Dimethylaminoethanol acts as a precursor to acetylcholine. It has been used in the treatment of hyperkinetic disorders and may lead to positive behavioral changes in patients with senile (73, 74). It has also skin-firming effects in cosmetology by increased contractility and adhesion in skin cells (75).

##### Ashwagandha (Withania somnifera)

This herb is used in Ayurvedic and Indian traditional medicine. It has been shown to have reasonable efficacy in the treatment of schizophrenia, chronic stress, insomnia, anxiety, memory and cognitive improvement, obsessive-compulsive disorder, rheumatoid arthritis, type-2 diabetes, and male infertility (76). Withanolide A, isolated from ashwagandha roots, is involved in neuritic regeneration and synaptic reconstruction. Thus, it may be an important candidate for the therapeutic treatment of neurodegenerative diseases (77, 78).

##### Blueberry (Vaccinium corymbosum) Extract

Antioxidants and polyphenols found in the blueberry extract have been shown to prevent a decline in synaptic strength and improve glutamate receptor function (79).

##### Ginkgo biloba

Ginkgo flavone glycosides and terpene lactones are considered to be the primary active components of *G*. *biloba* (80). The leaf extracts may have beneficial effects in treating several diseases such as neurodegenerative diseases, cardiovascular diseases, cancer, stress, memory loss, tinnitus, geriatric complaints such as vertigo, age-related macular degeneration, and psychiatric disorders like schizophrenia (81).

##### Eleuthero (Eleutherococcus senticosus)

It belongs to the ginseng family. It is suggested to have a stimulating effect on carbohydrate and fatty acid metabolism (82). Similar to ashwagandha, acute administration of eleuthero extract has been shown to strengthen short-term memory in healthy humans (83).

##### Vinpocetine

This is a derivative of vincamine, an alkaloid extracted from the lesser periwinkle plant. It acts as a vasodilator and may lead to cognitive enhancement and neuronal protection due to increased cerebral blood-flow (84). It is also used as a prescribed medicine for the treatment of dysfunctional cognitive abilities and cerebrovascular diseases (85).

##### Glycerophosphocholine

Glycerophosphocholine is a precursor of phosphatidylcholine, a source of choline where choline levels are linked to the rate of biosynthesis of acetylcholine (86, 87). It has shown to have a therapeutic role on the cognitive recovery of patients with an acute stroke (88). It was also reported to enhance muscle performance in humans (89).

##### Phosphatidylserine

This is a natural component of the cellular membranes, including in neurons. Its supplementation in humans has shown beneficial effects against age-associated memory loss (90).

### N247 Preparation

N247 formula is packed in 9 separate components: 2 brown capsules, 3 pinkish-tan capsules, 1 transparent soft gel, 1 maroon soft gel, 2 light yellow tablets. Soft gel capsules contain marine fish oil and lutein, tablets contain daily multivitamin, whereas the remaining components are included in the capsule. The following were added for increasing shelf life and delivery: cellulose coating, natural citrus flavor, natural lemon oil, rosemary extract, gelatin, glycerin, carboxymethylcellulose sodium, hydroxypropyl methylcellulose, cellulose, silicon dioxide, vegetable stearine, natural mixed tocopherol vitamin E, gum ghatti, magnesium stearate, stearic acid, and water. The product was formulated by Steven Osguthorpe and Dianne Osguthorpe (Optimal Health Research, Utah, USA) and produced by SupremeFill Nutraceuticals (Arizona, USA). After production, the shelf life is 3 years at room temperature.

### Subjects and Study Design

**Figure 1:**
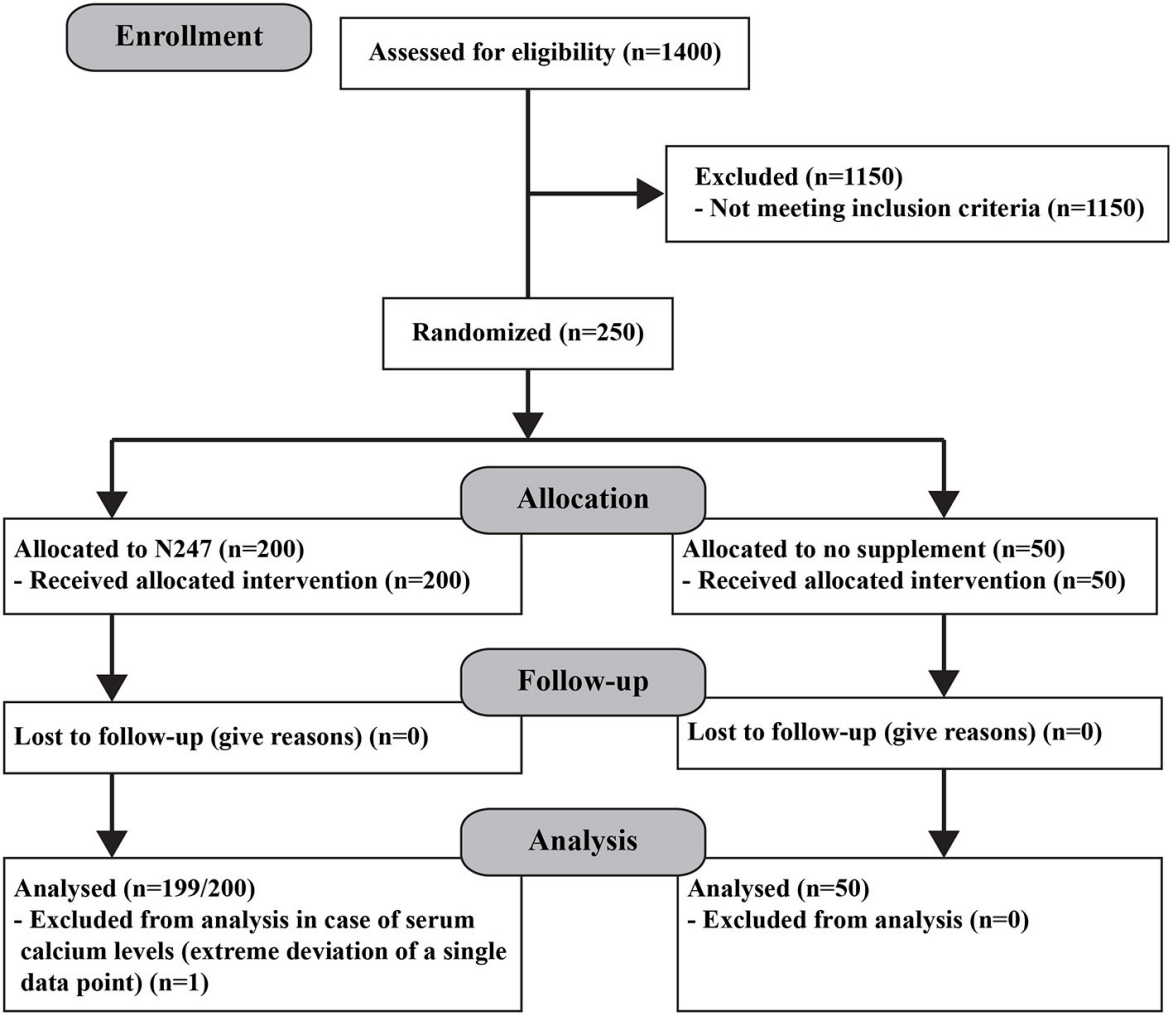
Consort diagram.

Two hundred and fifty subjects aged 26–75 were randomly selected using a random number generator from a pool of 1,400 who sought medical advice at the Optimal Health Clinic, Salt Lake City, Utah, USA. Subjects were in general health and did not have a long-term, debilitating disease/condition and/or taking prescription medication for it. They also had circulating serum vitamins A, B1, B2, B6, B12, C, D, E, calcium, iron, insulin-like growth factor 1 (IGF-1), and free triiodothyronine (FT-3) levels within normal levels. All subjects [117 women and 133 men (*N* = 250) from various ethnic backgrounds] provided written informed consent. Before enrolling in the study, the medical history of the subjects was registered and a physical examination was performed (Table 2). Subjects were then randomly divided into two groups, the control (*n* = 50; women = 24) and the N247 (*n* = 200; women = 93), using a random number generator; unequal randomization was used to decrease the cost of the trial. In addition, the study included a placebo control group (*n* = 35; women = 18) where the subjects ingested a preparation with similar visual characteristics to N247, but it contained rice flour in soft gel capsules and gelatin and rice flour in the tablets. All participants were processed by Optimal Health Research Star Valley, Wyoming USA, using the facilities of Lab Corp, Inc, and Optimal Health Clinic Inc., Salt Lake City, Utah, USA. The study was conducted between December 2008 and February 2009 and the follow-up of the study continued until April 2009. The study arm for the placebo control group was conducted from April–December 2020. A registered nurse followed the compliance. Subjects received a 1 month supply of N247 at the clinic when they arrived for follow-up; hence, the N247 group obtained supplies three times. N247 group took one packet of N247 once daily with breakfast. No one reported missing any dose. The control group did not take anything (Figure 1). The CONSORT checklist is presented as supporting information (Supplementary File 1).

**Table 2:** Subject characteristics.

	**Women**			**Men**		
	**N247**	**Control**	**Placebo**	**N247**	**Control**	**Placebo**
Total number (*N*)	93	24	18	107	26	17
**Ethnicity/race (** ***N*** **)**
White/Caucasian	60	15	11	68	17	12
Black/African American	11	3	3	13	3	2
Asian	4	1	1	4	1	0
Hispanic/Latino	15	4	2	17	4	2
Native American/PacificIslander	3	1	1	5	1	1
Age (years)	48.80 ± 15	50.1 ± 14.8	51.6 ± 15	51.7 ± 15.2	50.4 ± 14.1	53.2 ± 11.6
Age range (years)	26–75	28–75	28–74	29–75	26–73	30–73
Height (ft)	5.6 ± 0.3	5.7 ± 0.2	5.6 ± 0.2	6.0 ± 0.2	6.1 ± 1.1	6.0 ± 0.2
Weight (lb)	140 ± 13.4	153.4 ± 31.1	149.2 ± 12.2	190.4 ± 23.9	186.8 ± 11.2	192.9 ± 12.0
Systolic blood pressure (mmHg)	127.1 ± 9.6	129.0 ± 7.3	132.4 ± 6.9	125.0 ± 8.7	128.3 ± 8.1	129.6 ± 6.2
Diastolic blood pressure (mmHg)	82.7 ± 3.5	83.4 ± 3.6	82.4 ± 2.5	82.4 ± 3.3	82.4 ± 2.6	82.1 ± 3.3
Heart rate (bmp)	64.5 ± 2.9	69.2 ± 2.1	68.9 ± 2.1	61.4 ± 2.5	67.0 ± 3.3	66.2 ± 3.5

*Values are mean ± SD*.

The primary endpoints measured were the capability of N247 to increase the serum levels of the various vitamins and minerals compared with the control group at the end of the study. There were no drop-outs during the study and none of the participants reported any side effects of taking N247.

### Blood Sampling and Serum Preparation

Blood samples were collected at the beginning of the study serving as a baseline and then subsequently at 2 (only for the N247 group) and 3 months to determine serum levels of vitamins (A, C, D, E, B1, B2, B6, and B12) and minerals such as calcium and iron. IGF-1 and FT-3 were included to assess growth hormone and thyroid function.

Blood samples were collected and serum was prepared using standard methods by LabCorp (Salt Lake City, Utah, USA) and Quest Diagnostics (Utah, USA).

### Determination of Serum Vitamin and Mineral Levels

Serum vitamin and mineral levels were determined by standard methods. High-performance liquid chromatography was used to determine vitamin A, E, B1, and B12 levels in serum. Liquid chromatography tandem mass spectrometry (LC-MS/MS) was used to determine serum vitamin B6 levels. Capillary electrophoresis was used to quantify vitamin C levels and vitamin D levels were analyzed by LC-MS/MS. Arsenazo III method was used for the quantitative determination of total calcium in serum samples. Ferrozine method was used for quantitative analysis of serum iron levels. An immunochemiluminometric assay was used for quantitative assessment of serum IGF-1 levels. Serum FT-3 values were measured using a chemiluminescence assay.

### Data and Statistical Analysis

Collected data were screened for outliers. Analyses were carried out on IBM SPSS statistics for Macintosh, version 24 (SPSS Inc. Chicago, Ill., USA). Data on age and physiological measures of blood pressure, weight, and height, and serum levels for vitamins and minerals were normally distributed. Heart rate data were mildly skewed. Independent *t*-tests were performed to check for group differences in age and the physiological measures at baseline.

Serum vitamin and mineral levels, which were the dependent variables under investigation, were normally distributed, except for calcium which was moderately skewed for data at the end of 3 months. A single data point within the N247 group showed extreme deviation; hence, this was not considered for further analysis. Accordingly, for this outcome variable, the sample size for the N247 group was 249.

For exploratory purposes, paired *t*-tests were performed on the N247 group to compare serum levels at two-time points, *viz*, baseline and after 2 months of N247 intake, for men and women, separately. Data from control participants at 2 months were not available. The effect size *r* is given as √[*t*2/(*t*2 + df)]. A *p* < 0.05 was considered as significant. Confidence intervals were set at 99% to observe stringency.

Finally, separate 2 (treatment group–N247 and control) x 2 (time–baseline and after 3 months) mixed ANOVA tests were performed to check for main and interaction effects on each of the dependent variables. A *P* < 0.05 was considered statistically significant. If there were significant interaction effects in the mixed ANOVA output for within and between parameters of the subjects, tests of simple effects were calculated to check for significant differences of each pair of the dependent variable at each level of the between-subject variables. Mixed ANOVAs were the main analyses for the study; hence, *post-hoc* power analyses were based on repeated measures, within-between interaction ANOVA. Achieved power was computed using an α of 0.05, a sample size of 250, an average effect size of 0.50, and a non-sphericity correction *e* of 1.0; the obtained power was 0.99.

## Results

### Demographics and Baseline Measures

*T*-test results did not show differences between N247 and control groups for age, [*t*_(248)_ = 0.06, *p* = 0.9], height [*t*_(248)_ = −1.445, *p* = 0.1], and weight [*t*_(248)_ = −0.89, *p* = 0.38]. Group differences were present for systolic blood pressure readings [*t*_(248)_ = −2.00, *p* = 0.046, Cohen's *d* = 0.32] with the control group showing a higher reading (*M* = 128.64, *SD* = 7.64) than the N247 group (*M* = 126.05, *SD* = 8.32). However, diastolic blood pressure did not show significant group differences [*t*_(248)_ = −0.65, *p* = 0.52].

### Serum Changes in Vitamins and Minerals

**Figure 2:**
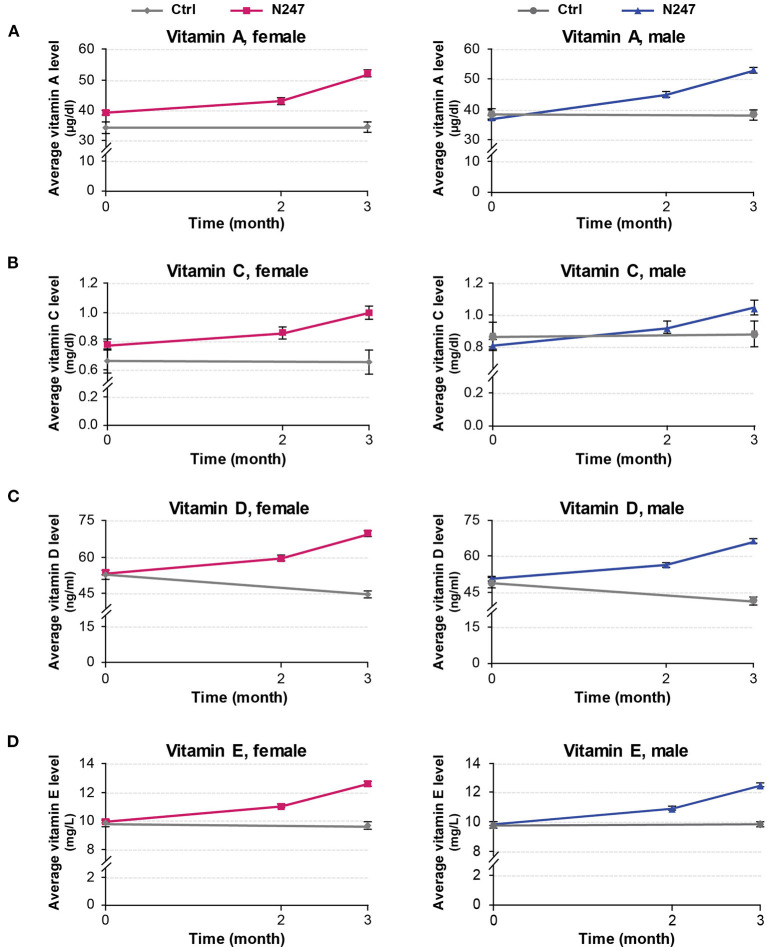
Graphical representation of N247 effects on serum vitamin A, C, D, and E levels. Serum concentrations of vitamin A **(A)**, vitamin C **(B)**, vitamin D **(C)**, and vitamin E **(D)** in control and N247 receiving group of women and men subjects during the study. Values are mean ± SEM.

**Figure 3:**
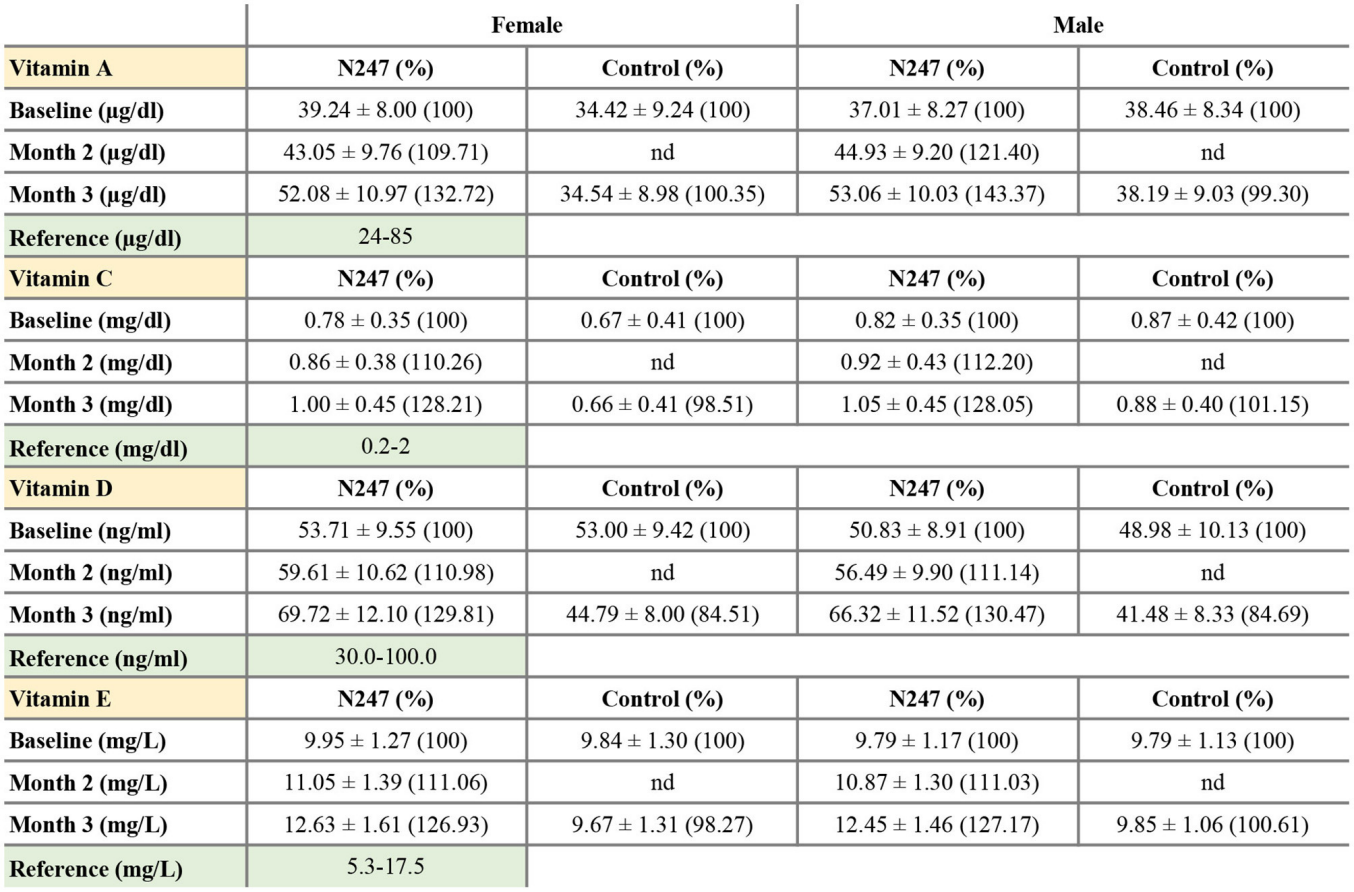
Serum concentrations of vitamin A, C, D, and E. Values are mean ± SD. % values are given in parentheses.

Men subjects had similar baseline levels of serum vitamin A in both groups (38.46 ± 8.34 μg/ml in the control group and 37.01 ± 8.27 μg/ml in the N247 group), whereas women subjects of N247 group had a higher baseline level compared with the control group (34.42 ± 9.24 μg/ml in the control group and 39.24 ± 8.00 μg/ml in N247 group). After 2 months of N247 consumption, serum vitamin A levels of both women and men subjects in the N247 group had a significant increase of 9.71 and 21.40%, respectively, compared with their baseline levels by paired *t*-test (Supplementary Table 1). At the end of 3 months, serum vitamin A levels of both women and men N247 groups increased to 32.72% in women and 43.37% in men, compared with the corresponding baseline values (Figures 2A, 3).

A 2 × 2 mixed ANOVA was performed with treatment groups (N247 and control) as the between variable of the subjects, and time (baseline and after 3 months) as the within variable of the subjects to examine the effect of N247 use on serum vitamin A levels within the time range of the study. There was a significant interaction effect between treatment groups and time [*F*_(1,248)_ = 61.30, *p* < 0.001, ηp^2^ = 0.20]. Test of simple main effects revealed a significant difference between N247 (*M* = 52.6, *SD* = 10.46) and control (*M* = 36.44, *SD* = 9.10) groups in vitamin A levels after 3 months [*F*_(1,248)_ = 100.21, *p* < 0.001]. A significant difference between baseline and 3 months for levels of vitamin A was found only for the N247 group [*F*_(1,248)_ = 303.14, *p* < 0.001]. There was a main effect of the group [*F*_(1,248)_ = 60.10, *p* < 0.001, ηp^2^ = 0.20] that showed a significant difference between the N247 and control groups (*p* < 0.001). Similarly, a main effect of time for vitamin A levels was also present [*F*_(1,248)_ = 59.96, *p* = 0.001, ηp^2^ = 0.20] with increased vitamin A levels after 3 months as compared with baseline (*p* < 0.001).

The vitamin C baseline levels of N247 and control groups were similar (0.87 ± 0.42 mg/dl in controls vs. 0.82 ± 0.35 mg/dl in N247 group in men; 0.67 ± 0.41 mg/dl in the control group and 0.78 ± 0.35 mg/dl in the N247 group in women). After 2 months of use of N247, there was a significant increase of 10.26% in the vitamin C levels of the N247 group compared with baseline in women, determined by paired *t*-test (Supplementary Table 1). After 3 months, there was still a significant increase (28.21%) in the N247 group, but a slight decrease of 1.49% in the control group. Similarly, vitamin C levels in men subjects were significantly increased both after 2 and 3 months of use of N247 compared with the baseline (12.20 and 28.05%, respectively) (Figures 2B, 3 and Supplementary Table 1).

A statistically significant interaction effect between treatment groups and time was present [*F*_(1,248)_ = 130.58, *p* < 0.001, ηp^2^ = 0.35]. Simple main effect analysis showed a significant difference between N247 (*M* = 0.77, *SD* = 0.41) and control (*M* = 1.03, *SD* = 0.45) groups in vitamin C levels after 3 months [*F*_(1,248)_ = 12.48, *p* < 0.001). A significant difference between baseline and 3 months was found in levels of vitamin C only for the N247 group [*F*_(1,248)_ = 676.65, *p* < 0.001].

The vitamin D baseline levels of N247 and control groups were similar (48.98 ± 10.13 ng/ml in controls vs. 50.83 ± 8.91 ng/ml in N247 group in men; 53.00 ± 9.43 ng/ml in controls vs 53.71 ± 9.55 ng/ml in N247 group in women). In the N247 group, there was a significant increase (10.98% in women and 11.14% in men) of serum vitamin D concentrations after 2 months of N247 consumption (Figure 2C and Supplementary Table 1).

The results of the 2 x 2 mixed ANOVA analysis revealed a significant interaction effect between treatment groups (N247 and control) and time (baseline and after 3 months) [*F*_(1,248)_ = 2,418.96, *p* < 0.001, ηp^2^ = 0.91]. There was a main effect of group [*F*_(1,248)_ = 64.50, *p* < 0.001, ηp^2^ = 0.21], with a higher level of vitamin D found in the N247 group than the control group (*p* < 0.001). There was also a main effect of time for vitamin D levels [*F*_(1,248)_ = 270.69, *p* < 0.001, ηp^2^ = 0.52], with higher levels after 3 months than at baseline (*p* < 0.001). Test of simple main effect analysis showed that the N247 group (*M* = 67.89, *SD* = 11.89) had significantly higher levels of vitamin D compared with the control group (*M* = 43.07, *SD* = 8.26) after 3 months [*F*_(1,248)_ = 194.40, *p* < 0.001]. Moreover, a significant increase between levels at baseline and 3 months for the N247 group was present [*F*_(1,248)_ = 5,384.25, *p* < 0.001]. In contrast, in the control group there was a significant decrease between levels at baseline and 3 months for the control group [*F*_(1,248)_ = 334.68, *p* < 0.001], which might be due to seasonal variance in sun exposure as the study was conducted during winter months (91–93).

The vitamin E baseline levels of N247 and control groups were similar (9.79 ± 1.13 mg/L in controls vs. 9.79 ± 1.17 mg/L in N247 group in men; 9.84 ± 1.30 mg/L in controls vs. 9.95 ± 1.27 mg/L in N247 group in women). Two months after N247 consumption, levels of vitamin E significantly increased (11.06% for women and 11.03% for men) compared with baseline, determined by paired *t*-test (Supplementary Table 1), which further increased after 3 months (26.93% in women and 27.17% in men compared with baseline) (Figures 2D, 3).

A significant interaction effect between treatment groups and time was present [*F*_(1,248)_ = 1,716.28, *p* < 0.001, ηp^2^ = 0.87] determined by two-way mixed ANOVA. Test of simple main effects revealed a significant difference between N247 (*M* =12.53, *SD* = 1.53) and control (*M* = 9.76, *SD* = 1.18) groups in vitamin E levels after 3 months [*F*_(1,248)_ = 142.59, *p* = 0.001]. A significant difference was present in the levels between baseline and after 3 months for the N247 group [*F*_(1,248)_ = 8,207.07, *p* < 0.001].

**Figure 4:**
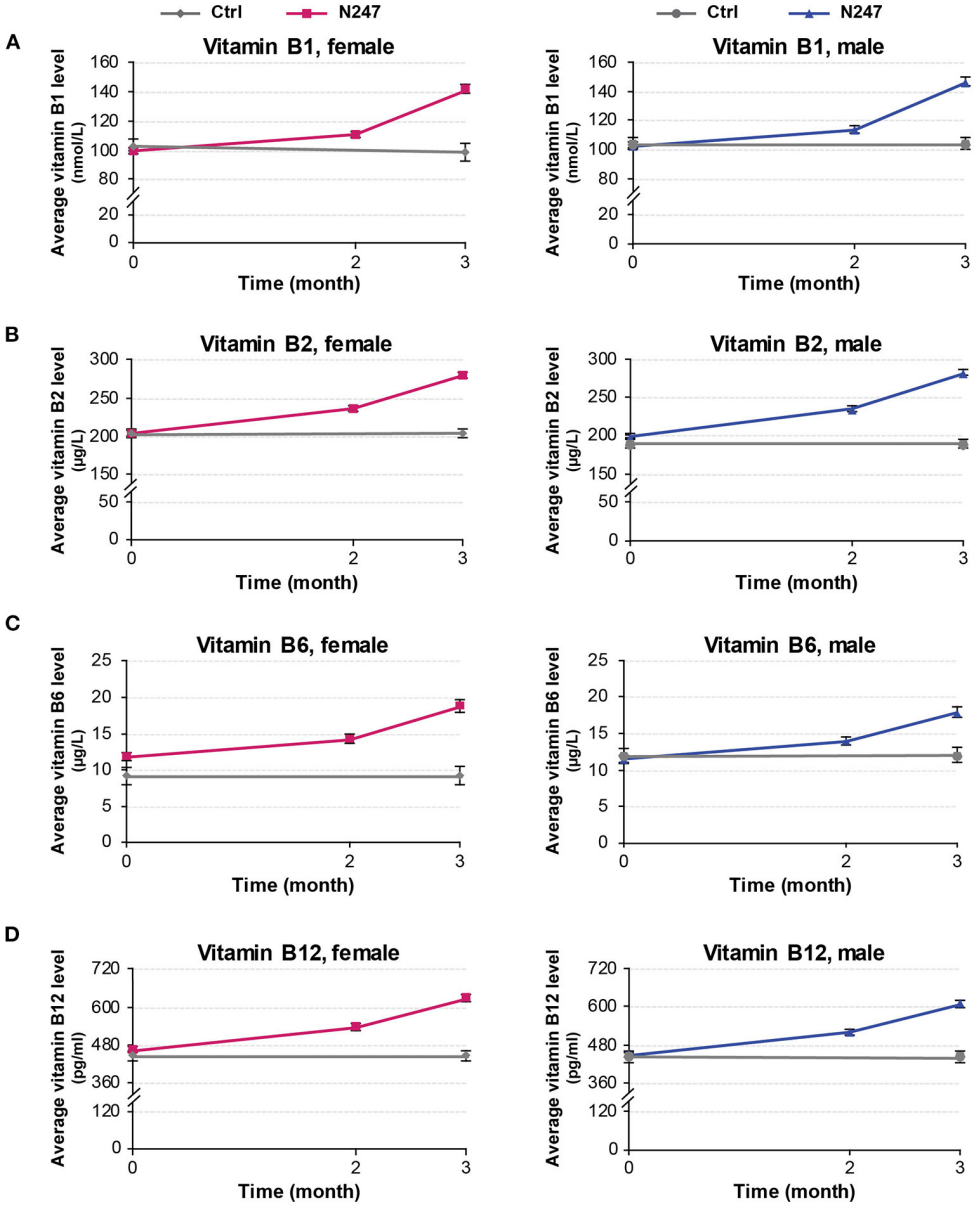
Graphical representation of N247 effects on serum vitamin B1, B2, B6, and B12 levels. Serum concentrations of vitamin B1 **(A)**, vitamin B2 **(B)**, vitamin B6 **(C)**, and vitamin B12 **(D)** in control and N247 receiving group of women and men subjects during the study. Values are mean ± SEM.

**Figure 5:**
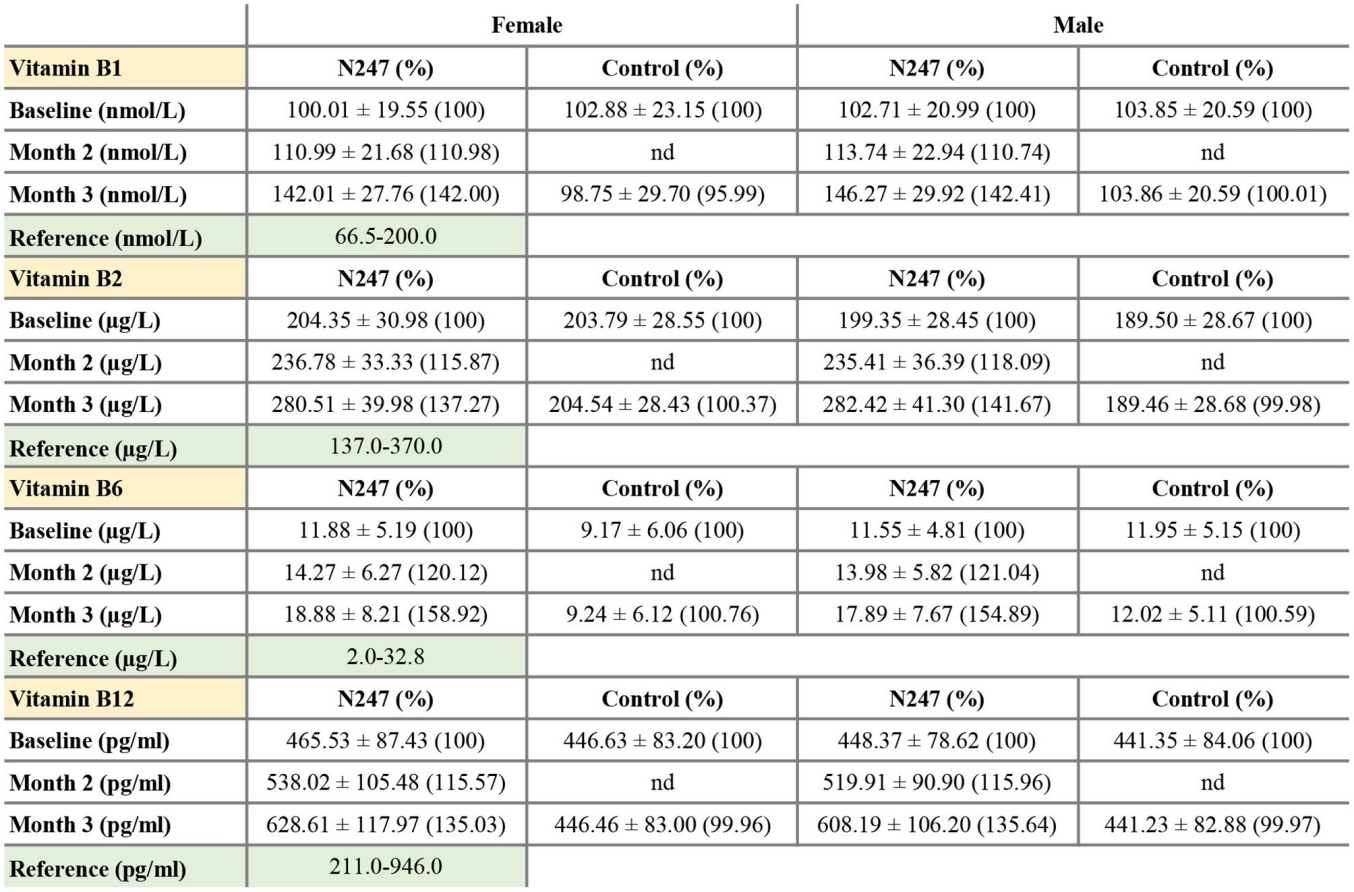
Serum concentrations of vitamin B1, B2, B6, and B12. Values are mean ± SD% values are given in parentheses.

For both genders, the baseline levels of vitamin B1, B2, B6, and B12 of N247 and control groups were similar (Figures 4, 5), although for females the B6 vitamin baseline levels for N247 and control were slightly, but significantly different (Figure 4C). For the N247 group, serum vitamin B1, B2, B6, and B12 levels were significantly increased for both genders (10.98, 15.87, 21.04, and 15.57% for women, and 10.74, 18.09, 20.12, and 15.96% for men, respectively) compared with baseline after 2 months use of N247, which was further increased at 3 months (Supplementary Table 1 and Figure 5).

The 2 × 2 mixed ANOVA analysis revealed a significant interaction between groups (N247 and control) and time (baseline and after 3 months) on the serum concentrations of vitamin B (Supplementary Table 2). For all the investigated B vitamins, there was a main effect of group with higher levels of B vitamins in the N247 group than the control group [for vitamin B1 *F*_(1,248)_ = 28.64, *p* < 0.001, $\eta_{\text{p}}^{2}$ = 0.10; for vitamin B2 *F*_(1,248)_ = 131.10, *p* < 0.001, $\eta_{\text{p}}^{2}$ = 0.35; for vitamin B6 *F*_(1,248)_ = 19.43, *p* < 0.001, $\eta_{\text{p}}^{2}$ = 0.07, and for vitamin B12 *F*_(1,248)_ = 38.92, *p* < 0.001, $\eta_{\text{p}}^{2}$ = 0.14; as shown in Supplementary Table 2]. There was also a main effect of time with higher levels of B vitamins at 3 months of study than the baseline [for vitamin B1 *F*_(1,248)_ = 657.75, *p* < 0.001, $\eta_{\text{p}}^{2}$ = 0.73; for vitamin B2 *F*_(1,248)_ = 133.25, *p* < 0.001, $\eta_{\text{p}}^{2}$ = 0.35; for vitamin B6 *F*_(1,248)_ = 204.71, *p* < 0.001, $\eta_{\text{p}}^{2}$ = 0.45, and for vitamin B12 *F*_(1,248)_ = 1,246.78, *p* < 0.001, $\eta_{\text{p}}^{2}$ = 0.83; as shown in Supplementary Table 2]. Test of simple effects showed that concentration of B vitamins was significantly increased in N247 group compared with control group after 3 months (Supplementary Table 2). Moreover, the level of B vitamins significantly differed at baseline and after 3 months in the N247 group (Supplementary Table 2).

**Figure 6:**
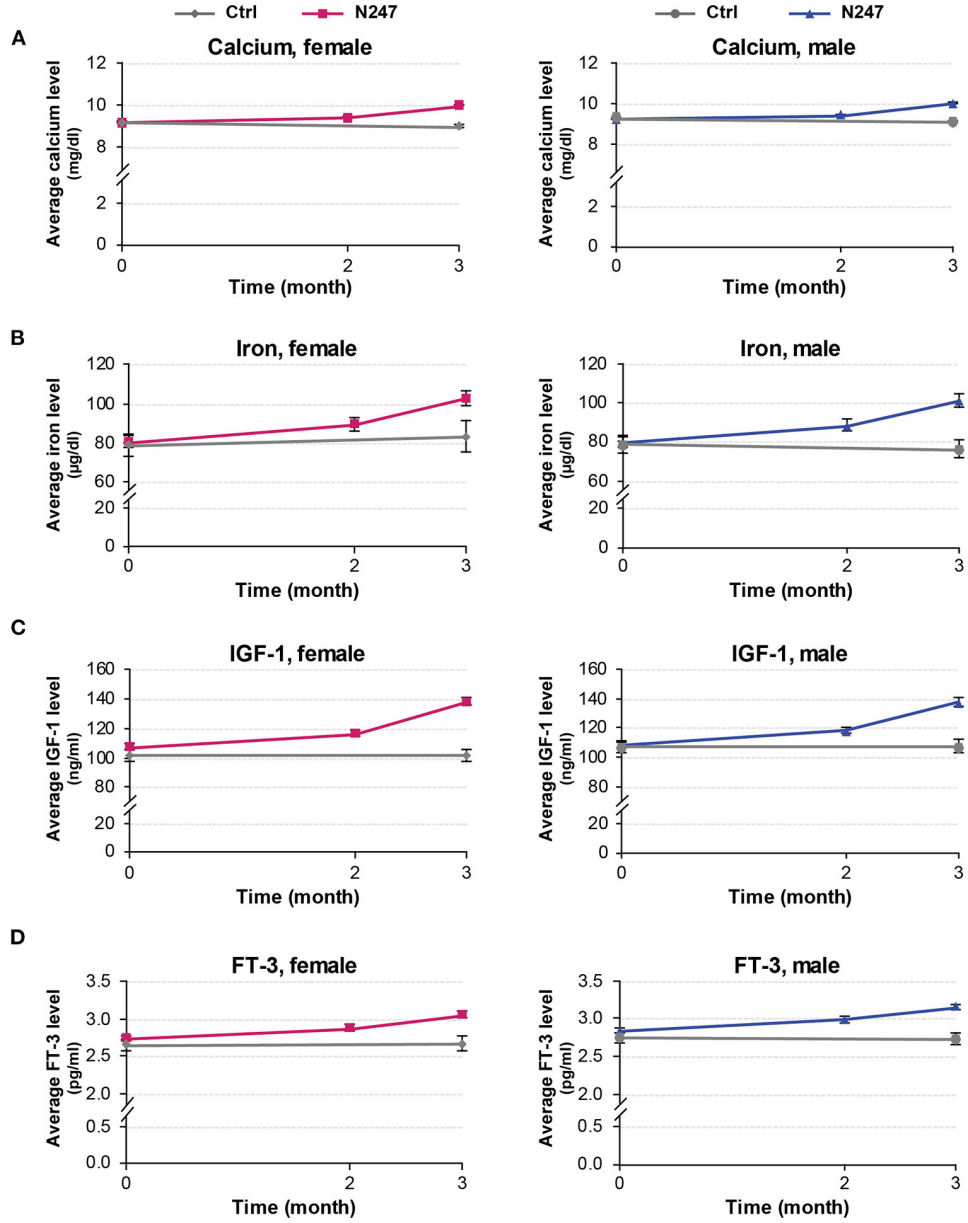
Graphical representation of N247 effects on serum calcium, iron, IGF-1, and FT-3 levels. Serum concentrations of calcium **(A)**, iron **(B)**, IGF-1 **(C)**, and FT-3 **(D)** in control and N247 receiving group of women and men subjects during the study of 3 months. Values are mean ± SEM.

**Figure 7:**
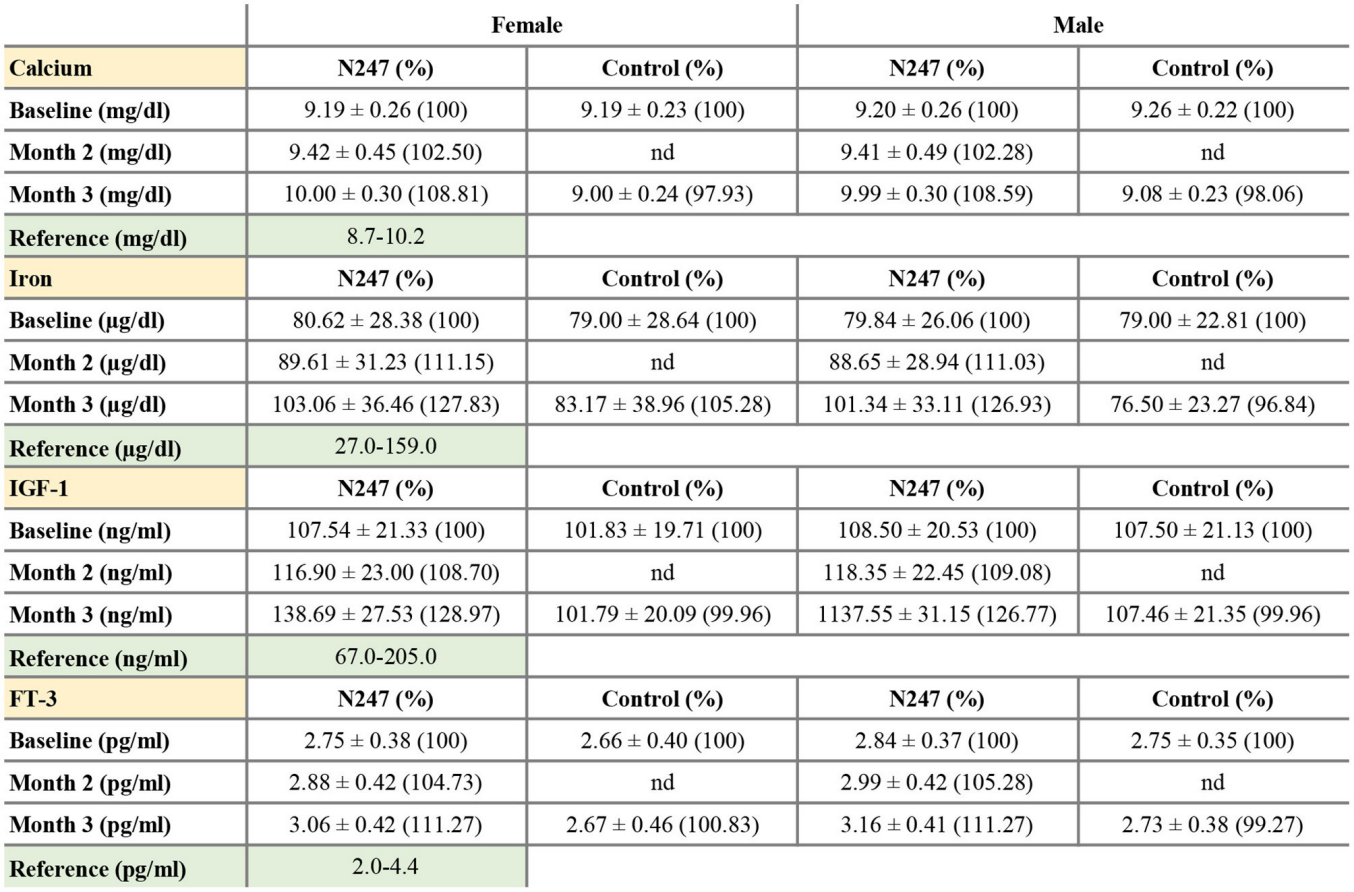
Serum concentrations of calcium, iron, IGF-1, and FT-3. Values are mean ± SD% values are given in parentheses.

The baseline concentrations of calcium in N247 and control groups were similar (9.26 ± 0.22 mg/dl in controls vs 9.20 ± 0.26 mg/dl in N247 group in men; 9.19 ± 0.23 mg/dl in controls vs. 9.19 ± 0.26 mg/dl in N247 group in women). Serum calcium levels were significantly increased with 2.50% and 2.28% compared with baseline after 2 months of N247 consumption in women and men subjects, respectively, which further increased after 3 months to 8.81 and 8.59% compared with baseline in women and men, respectively (Figures 6A, 7 and Supplementary Table 1). On the contrary, calcium levels decreased in the control group during the same time period (2.07% and 1.94% compared with baseline in women and men subjects, respectively). This may potentially be due to seasonal influences as previously reported (94).

The two-way mixed ANOVA analysis showed a significant interaction effect between treatment groups and time [*F*_(1,247)_ = 6,686.25, *p* < 0.001, ηp^2^ = 0.86]. Simple main effects analysis revealed a significant difference between N247 (*M* = 10.01, *SD* = 0.30) and control (*M* = 9.04, *SD* = 0.23) groups in calcium levels after 3 months [*F*_(1,247)_ = 439.13, *p* < 0.001]. Furthermore, serum calcium concentration was significantly upregulated at the 3-month time point compared with the baseline in the N247 group [*F*_(1,247)_ = 2,2215.94, *p* < 0.001], whereas it was significantly downregulated in the control group [*F*_(1,247)_ = 280.73, *p* < 0.001].

In both genders, serum iron baseline of N247 and control group of subjects were similar (79.00 ± 22.81 μg/dl in the control group and 79.84 ± 26.06 μg/dl in N247 group in men; 79.00 ± 28.64 μg/dl in the control group and 80.62 ± 28.38 μg/dl in N247 group in women). After 2 months of N247 consumption, levels of iron significantly increased in the N247 group of subjects (11.15% for women and 11.03% for men) compared with baseline (Figures 6B, 7 and Supplementary Table 1). Furthermore, the increase was greater after 3 months of use of N247 (27.83% in women and 26.93% in men compared to baseline). In the control groups, there was a difference of 5.28% and 3.16% in serum iron levels of women and men subjects, respectively during the study (Figures 6B, 7).

A significant interaction effect between treatment groups and time was found by two-way mixed ANOVA test [*F*_(1,248)_ = 153.78, *p* < 0.001, ηp^2^ = 0.38]. Simple main effect analysis showed a significant difference between N247 (*M* = 102.14, *SD* = 34.63) and control (*M* = 79.70, *SD* = 31.62) groups in serum iron levels after 3 months [F_(1,248)_ = 17.36, *p* < 0.001]. A significant difference between iron levels at baseline and 3 months for the N247 group was also observed [*F*_(1,248)_ = 820.41, *p* < 0.001].

Serum baseline IGF-1 concentrations of N247 and control groups were similar in both genders (2.75 ± 0.35 pg/ml in controls vs. 2.84 ± 0.37 pg/ml in N247 group in men; 2.66 ± 0.40 pg/ml in controls vs. 2.75 ± 0.38 pg/ml in N247 group in women). Mean IGF-1 serum concentration significantly rose 2 months after N247 consumption (8.70 and 9.08% increase compared to baseline in women and men, respectively) determined by paired *t*-test, which increased with 28.97 and 26.77% at 3 months in women and men subjects, respectively (Figures 6C, 7 and Supplementary Table 1).

The results of the 2 × 2 mixed ANOVA showed that there was a significant interaction effect between treatment groups and time [*F*_(1,248)_ = 246.74, *p* < 0.001, ηp^2^ = 0.50]. Test of simple main effects presented a significant difference between N247 (*M* = 138.08, *SD* = 29.45) and control (*M* = 104.74, *SD* = 20.74) groups in IGF-1 levels after 3 months [*F*_(1,248)_ = 56.92, *p* < 0.001], and a significant difference between IGF-1 levels at baseline and 3 months for the N247 group [*F*_(1,248)_ = 1,230.42, *p* < 0.001].

Baseline concentrations of serum FT-3 in N247 and control groups were similar for both genders (2.75 ± 0.35 pg/ml in controls vs. 2.84 ± 0.37 pg/ml in N247 group in men; 2.66 ± 0.4 pg/ml in controls vs. 2.75±0.38 pg/ml in N247 group in women). Mean FT-3 concentration significantly increased after 2 months of use of N247 (4.73 and 5.28% of increase compared with baseline in women and men, respectively) (Figures 6D, 7 and Supplementary Table 1). After 3 months of N247 consumption, mean serum FT-3 concentrations were elevated with 11.27% compared with baseline in both genders, whereas mean serum FT-3 concentrations were at similar levels during the study.

The two-way mixed ANOVA test showed a significant interaction effect between treatment groups and time [*F*_(1,248)_ = 421.43, *p* < 0.001, ηp^2^ = 0.63]. Moreover, simple main effect tests presented a significant difference between N247 (*M* = 3.11, *SD* = 0.42) and control (*M* = 2.70, *SD* = 0.42) groups in FT-3 levels after 3 months [*F*_(1,248)_ = 38.35, *p* < 0.001], whereas a significant difference was found between FT-3 levels at baseline and 3 months for the N247 group [*F*_(1,248)_ = 2,054.31, *p* < 0.001].

Placebo group: For the subjects that took the placebo formulation, the levels of different vitamins and minerals in their circulation were overall similar to those in the control group during the study (Supplementary Figures 1–3). Some statistically significant declines in specific vitamins and minerals were observed, for example, for Vitamin D, Vitamin C, and calcium, which were also observed in the control group, these changes were marginal and likely due to seasonal variations.

To determine whether N247 has differential effects in subjects of different ages, we categorized the data by three age groups, that is, 26–40, 41–55, and 56–75 years for the N247 group and repeated the analyses. As shown in Supplementary Figure 4, there was a significant change between baseline and 3 months for all vitamins and minerals in all the age groups. The details of these findings are presented in Supplementary Table 4. These data suggest that the changes in vitamin and mineral levels induced by N247 consumption are independent of age.

Altogether, these data document that the changes in circulating vitamin and mineral levels observed in the N247 group were specific to the formulation.

## Discussion

Dietary multivitamin/mineral supplement use is at an all-time high where they are readily available in pharmacies, health food stores, and online. However, there is very limited information on how effective these supplements are, that is, most of the supplements are not studied for their efficacy. The goal of this randomized controlled trial was to test whether regular consumption of a novel vitamin, mineral, and nutrient formula can efficiently increase serum levels of vitamins and minerals in healthy subjects. The data show that regular consumption of N247 for 3 months, which contains not only vitamins and minerals, but also other key nutrients, results in a statistically significant increase in serum concentrations of vitamins A, C, D, E, B1, B2, B6, B12, and calcium, iron, IGF-1, and FT-3. Moreover, serum levels of these vitamins and minerals were further increased but not over the reference range upper limits, at 3 months of use of N247.

Interestingly, serum levels of vitamin D in the control group significantly decreased during the study period, whereas they were significantly elevated in the N247 group. The decrease of mean vitamin D concentration in the control group maybe due to low exposure to sunlight as the study was carried out from December to April (91–93). Furthermore, there was a significant decline in serum calcium concentrations in the control group during the trial, which is also likely a direct result of lack of sun exposure (95).

The unique formulation of N247 offers a broader variety of ingredients in addition to vitamins and minerals compared to the common over-the-counter multivitamin/mineral supplements (Supplementary Table 3 and N247 ingredient profile). For example, N247 contains acetyl-l-carnitine, ashwagandha, coenzyme Q10, Rhodiola root, etc., which are expected to increase the effectiveness of the vitamins and minerals, as well as bringing additional benefits to increase wellness.

The source and the number of active ingredients are important determinants for the effectiveness and lack of toxicity of the dietary supplements, as well as authenticating them; however, for most common multivitamin/mineral supplements the active ingredients, their source (natural or synthetic), and the amounts are not listed in detail, and thus it is difficult to evaluate them from this angle. This is important, as the establishment of quality and safety of supplements is a critical point in limiting any potential adverse health effects (96). N247 is an example of how such a supplement should have documentation.

A unique aspect of the N247 formulation is that it also includes components that fight against aging and nervous system decline. An increase in the aging population around the world leads to a growing number of age-related diseases, such as a decrease in brain microcirculation, which may lead to Alzheimer's disease, changes in neurotransmitter production and function, hormonal imbalances related to chronic stress, cellular damage caused by free radicals in the brain, and metabolism which may cause diabetes and obesity (97). Thus, the aging population brings health and social challenges that need to be addressed worldwide. Controlled studies with preparations of N247 with and without these additional nutrients are necessary to test for its possible beneficial effects on the nervous system in general and in the aging population in particular.

Cardiovascular disease has an impact on millions of people in the United States and Europe (e.g., one of five Americans have a condition of high blood pressure) and remains the leading cause of death worldwide (98, 99). The principle reasons behind this deadly disease can be greatly ameliorated with the right diet and exercise regimen (100). Several supplements prevented heart conditions in clinical studies and are used therapeutically to treat certain heart ailments. For example, magnesium homeostasis is associated with better survival in chronic kidney disease cohorts (101). Selenium and coenzyme Q10 supplementation in the elderly have been linked to reduced cardiovascular mortality (102, 103). As N247 contains several key components, it can have a beneficial impact on cardiovascular health.

### Limitations and Future Directions

It would have been desirable to have an equal distribution of subjects in the two groups, N247 and control, and the placebo group. Since the differences between the groups that we observe are highly significant, we feel that similar results would be obtained with this modification in place. Future studies will need to address this point.

The findings establish that N247 efficiently increases serum levels of essential vitamins and minerals in subjects of a diverse age range. Coupled with other ingredients that are known to support the different organ systems, N247 may represent a novel and effective supplement to avoid nutritional deficiencies for better health and wellness.

## Data Availability Statement

The original contributions presented in the study are included in the article/Supplementary Material, further inquiries can be directed to the corresponding author/s.

## Ethics Statement

The studies involving human participants were reviewed and approved by Optimal Health Research IRB #3 US National Institutes of Health. The patients/participants provided their written informed consent to participate in this study.

## Author Contributions

SO conceptualized, designed the study, and collected the raw data. AS and HN conducted the statistical analysis. AS, HN, and FS interpreted the data and together with SO drafted the article. All the authors significantly edited and approved the article.

## Conflict of Interest

SO is employed by the company Optimal Health Research. SO is married to Dianne Osguthorpe, the owner of ReaLifeResources, Inc, which is the owner of N247, the subject matter of this manuscript. The remaining authors declare that the research was conducted in the absence of any commercial or financial relationships that could be construed as a potential conflict of interest.

## Publisher's Note

All claims expressed in this article are solely those of the authors and do not necessarily represent those of their affiliated organizations, or those of the publisher, the editors and the reviewers. Any product that may be evaluated in this article, or claim that may be made by its manufacturer, is not guaranteed or endorsed by the publisher.
