# Supplementary material for: A Nutraceutical Formula Is Effective in Raising the Circulating Vitamin and Mineral Levels in Healthy Subjects: A Randomized Trial
Source: Front Nutr. 2021 Sep 1;8:703394. doi: 10.3389/fnut.2021.703394 (PMC8440802; doi:10.3389/fnut.2021.703394)
Supplement: Supplementary file 6 [file Data_Sheet_2.pdf]

Supplementary Material

Supplementary File 1: CONSORT Checklist

Supplementary Table 1: Paired t test comparing baseline concentrations of serum vitamin and mineral vs. concentrations after 3 months of N247 consumption. t: t value; df: degree of freedom; CI: confidence interval; p: significance (2-tailed); r: effect size

Supplementary Table 2: Statistic values of two-way mixed ANOVA test for vitamin B1, B2, B6 and B12. F: F value; p: significance; η_p^2: partial eta squared; M±SD: mean ± standard deviation

Supplementary Table 3: Comparison of ingredient lists and dosages of N247 with two other dietary supplement brands. [*]% Daily value not established.

Supplementary Table 4: Statistical analysis comparing baseline concentrations of serum vitamin and minerals vs. values after 3 months of N247 consumption for the different age groups. M: mean; SD: standard deviation; p: significance

Supplementary Figure 1: Graphical representation of placebo effects on serum vitamin A, C, D and E levels. Serum concentrations of vitamin A **(A)**, vitamin C **(B)**, vitamin D **(C)** and vitamin E **(D)** in placebo group of female and male subjects during the study. Values are mean ± SEM.

Supplementary Figure 2: Graphical representation of placebo effects on serum vitamin B1, B2, B6 and B12 levels. Serum concentrations of vitamin B1 **(A)**, vitamin B2 **(B)**, vitamin B6 **(C)** and vitamin B12 **(D)** in placebo group of female and male subjects during the study. Values are mean ± SEM.

Supplementary Figure 3: Graphical representation of placebo effects on serum calcium, iron, IGF-1 and FT-3 levels. Serum concentrations of calcium **(A)**, iron **(B)**, IGF-1 **(C)** and FT-3 **(D)** in placebo group of female and male subjects during the study. Values are mean ± SEM.

Supplementary Figure 4: Graphical representation of N247 effects on serum vitamin and mineral levels for different age groups. Serum concentrations for the age group of 26-40 years **(A)**, 41-55 years **(B)**, 56-75 years **(C)**. Values are mean ± SD.
