# Supplementary figures and images for: A Nutraceutical Formula Is Effective in Raising the Circulating Vitamin and Mineral Levels in Healthy Subjects: A Randomized Trial
Source: Front Nutr. 2021 Sep 1;8:703394. doi: 10.3389/fnut.2021.703394 (PMC8440802; doi:10.3389/fnut.2021.703394)

Suppl. Figure 1

A)

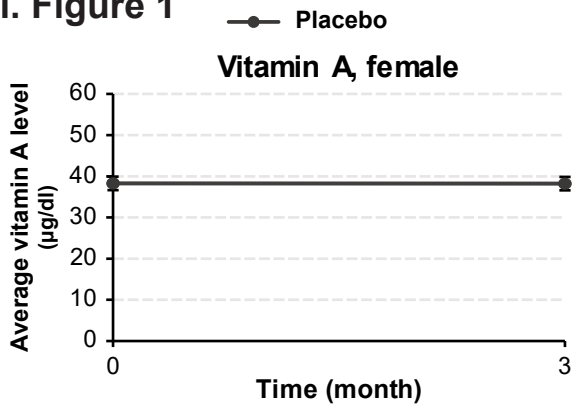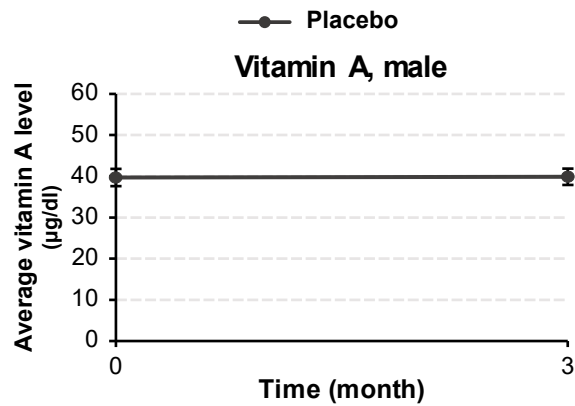

B)

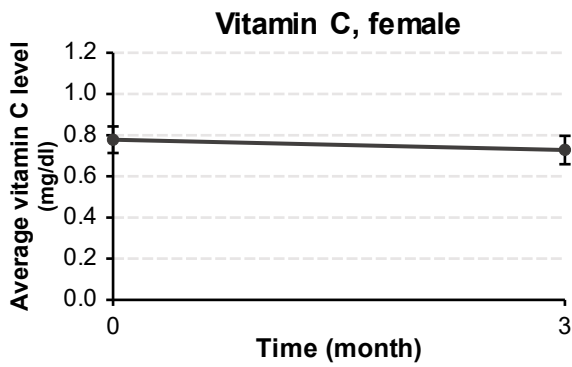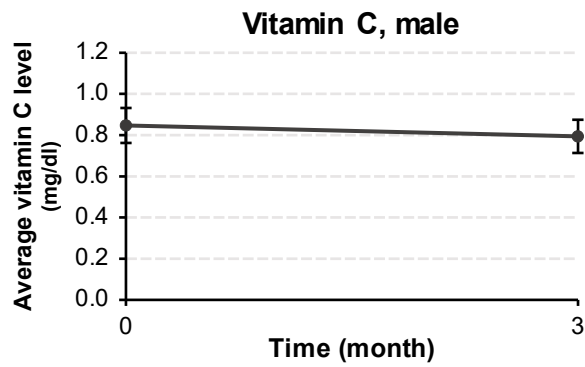

C)

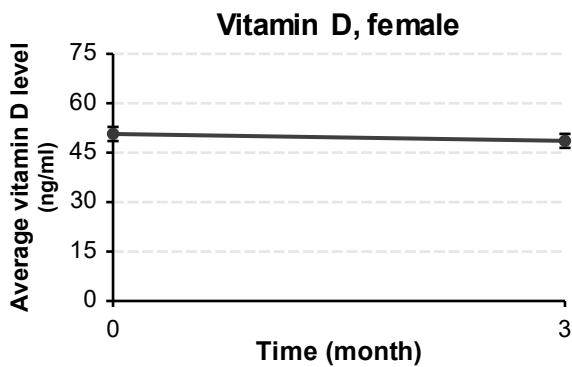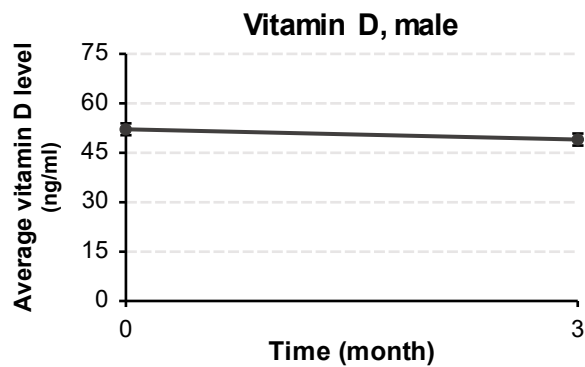

D)

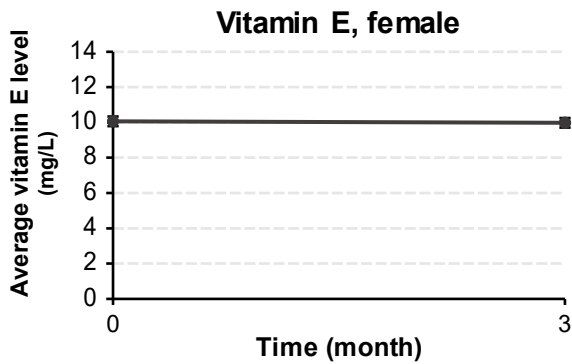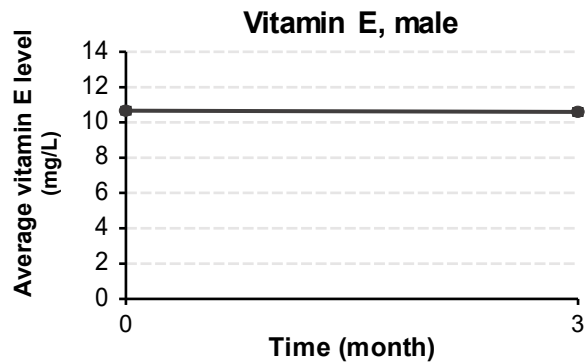

Supplement: Supplementary file 7 [file Image_1.pdf]

Suppl. Figure 2

A)

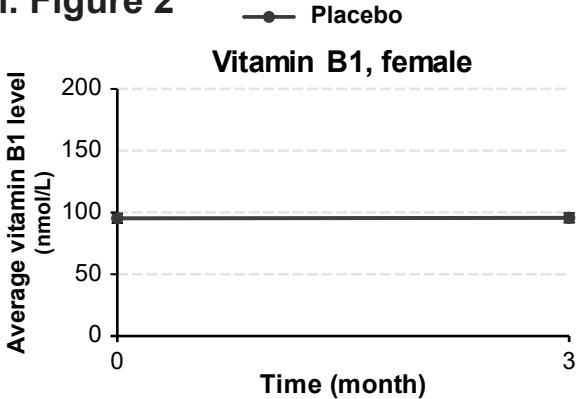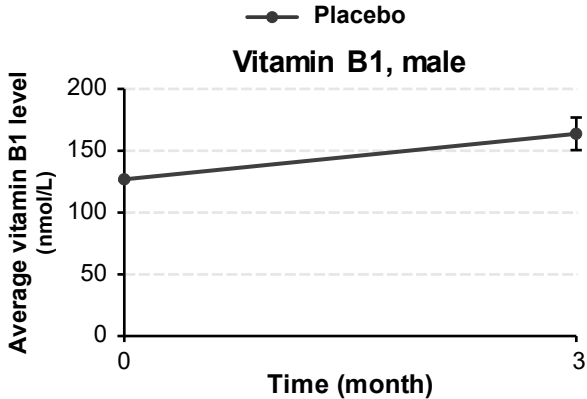

B)

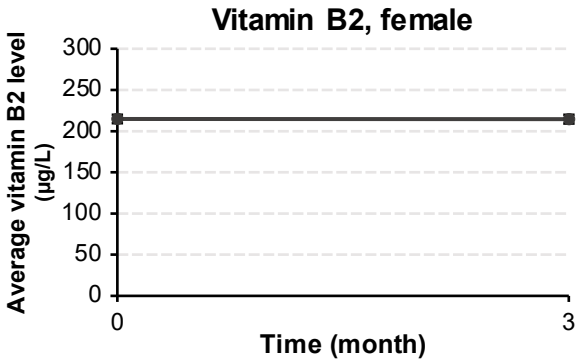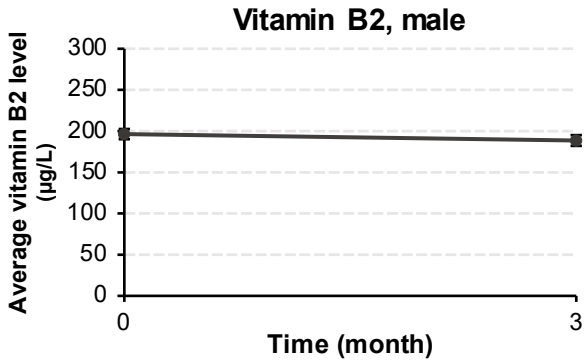

C)

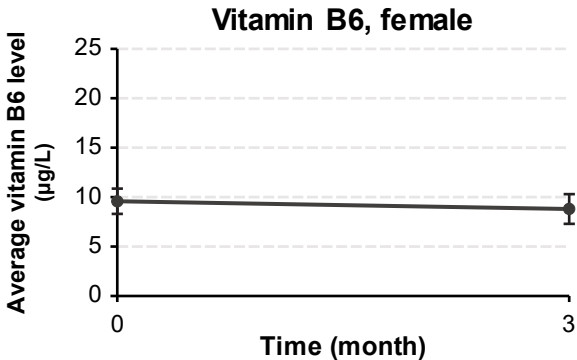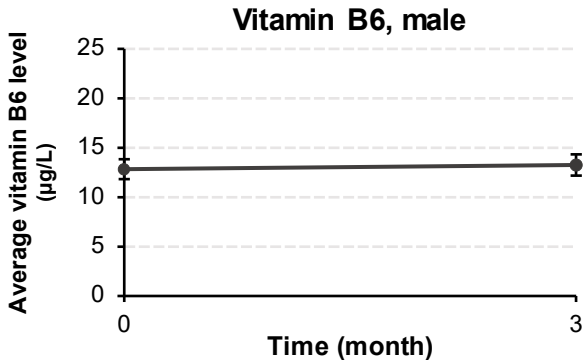

D)

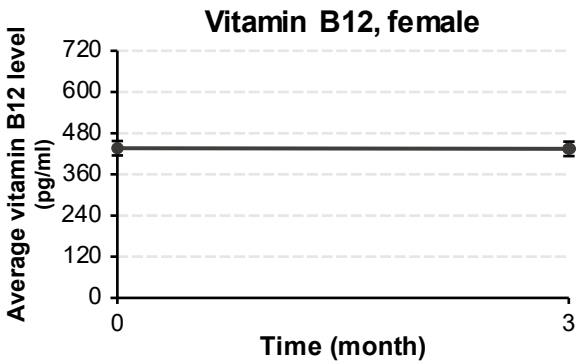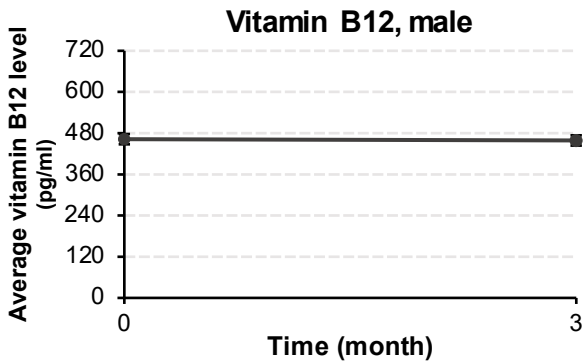

Supplement: Supplementary file 8 [file Image_2.pdf]

Suppl. Figure 3

A)

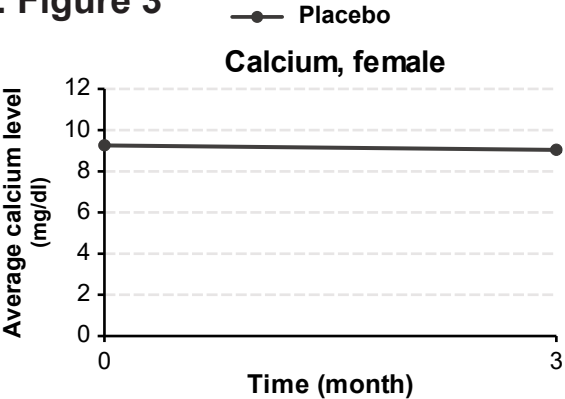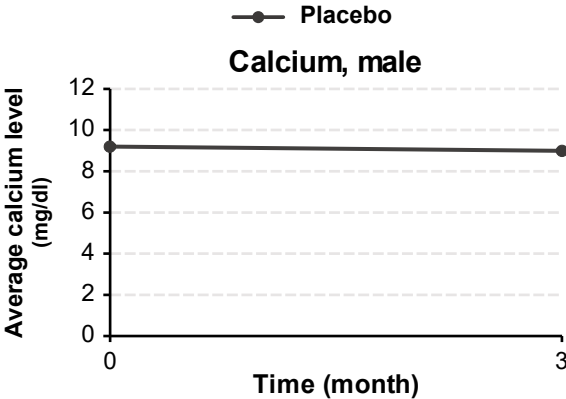

B)

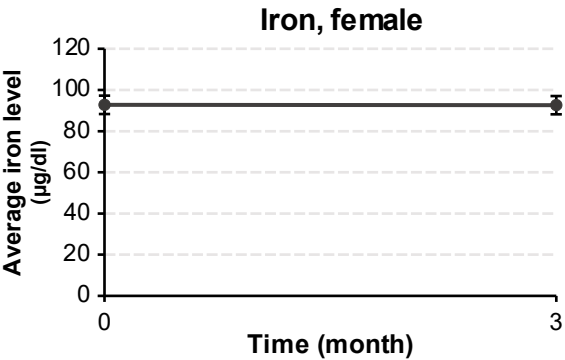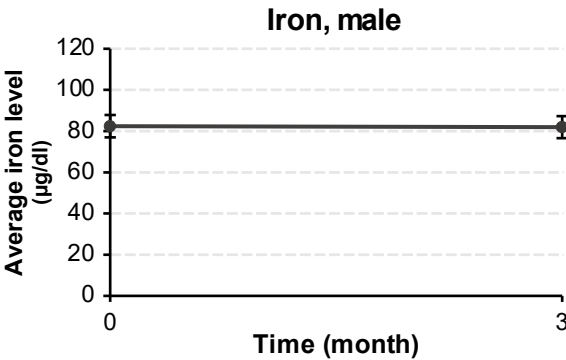

C)

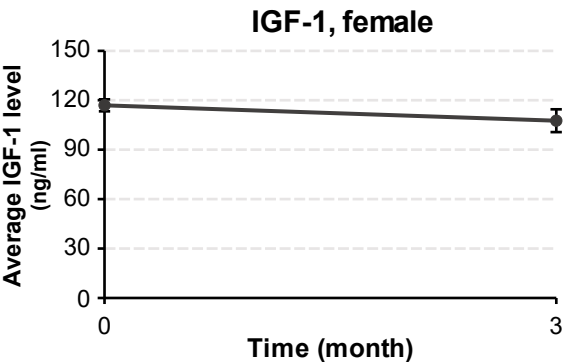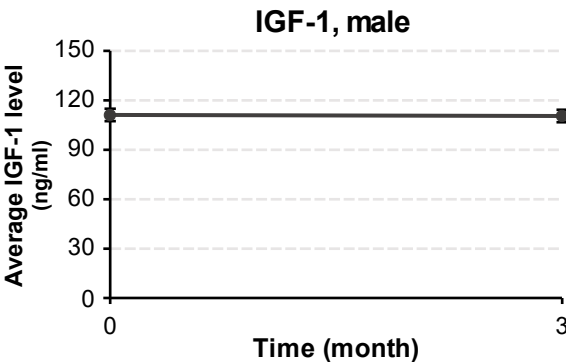

D)

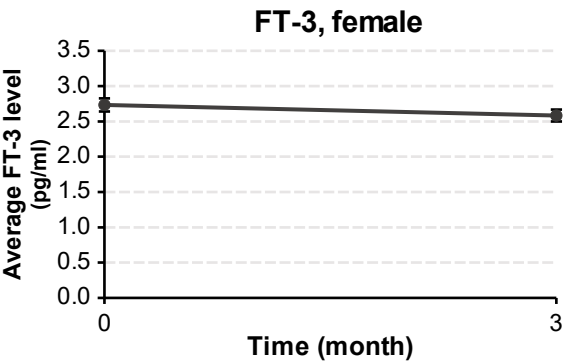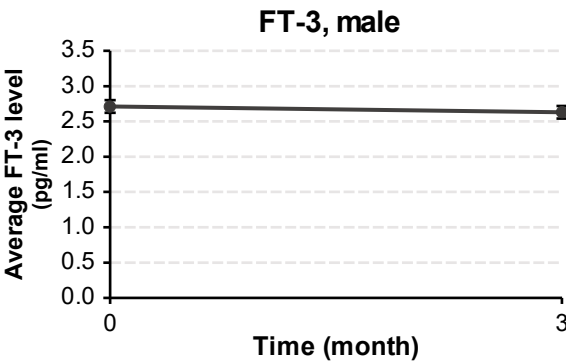

Supplement: Supplementary file 9 [file Image_3.pdf]

Suppl. Figure 4

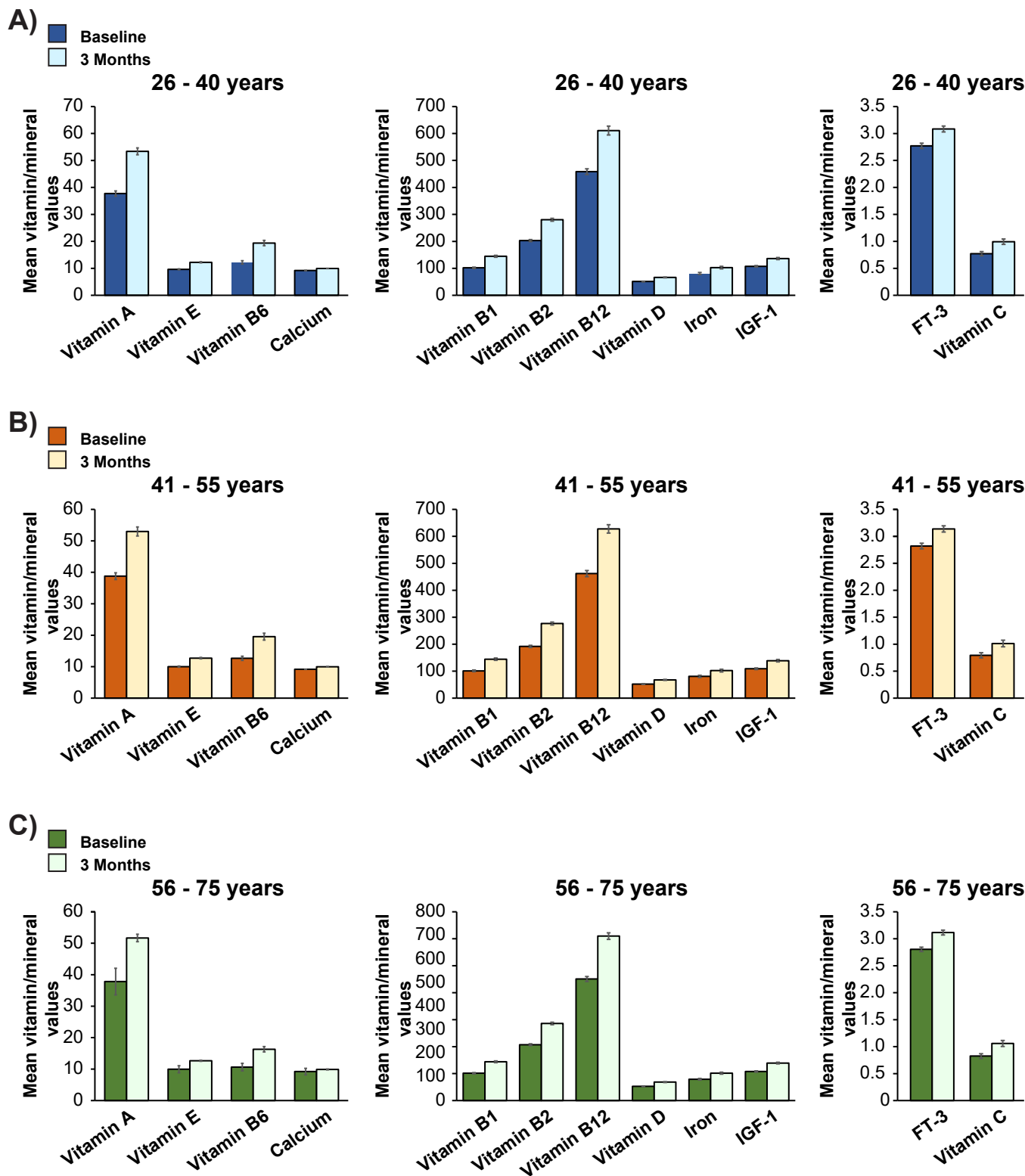

Supplement: Supplementary file 10 [file Image_4.pdf]
